# Survey of off-label anti-IL1 treatments in France: preliminary data

**DOI:** 10.1186/1546-0096-9-S1-P20

**Published:** 2011-09-14

**Authors:** L Rossi-Semerano, B Fautrel, C Galeotti, I Koné-Paut

**Affiliations:** 1 Department of Paediatrics and Paediatric Rheumatology, Hôpital de Bicêtre, National Reference Centre for Auto-inflammatory Diseases, Le Kremlin-Bicêtre, France; 2 Department of Rheumatology, Hôpital La Pitié Salpêtrière, Paris, France

## Background

Despite their limited licensed indications, anti-IL1 agents are often used in real-life practice for an increasing number of diseases. A national survey to record their off-label use in France was started in January 2011. The survey is coordinated by the French National Reference Centre for Auto-Inflammatory Diseases, under the aegis of the “Club Rhumatisme et Inflammation” (CRI).

## Aims

The survey aims at gathering information concerning: the number of patients treated with anti-IL1 agents in France, the treated disease, the kind and the indication of the used anti-IL1 agents, their efficacy and safety.

## Methods

We set up a physician-directed questionnaire available on the website of CRI since January 2011 to December 2012, covering the following areas: patient data, disease data, anti-IL1 agent, its efficacy, adverse events.

We advertised the study on the occasion of French rheumatology congresses and by email to French physicians that could be interested.

Any adult or paediatric patient who had received an anti-IL1 agent before January 2005 in France could be included.

## Results

At present 94 patients from 19 centres have been included. Main diseases were: anakinra-treated CAPS (18), SoJIA (17), AoSD (15), gout (10), MKD (9), FMF (6), Schnitzler’s syndrome (5).

The main off-label used agent (data known for 72 patients) was anakinra, used at least once in 66 patients. Canakinumab was used in 15 patients. Rilonacept is not yet available in France.

## Conclusions

Data gathering on off-label use of anti-IL1 agents is ongoing in France, with a good response from physicians providing information from everyday practice.

The survey results might justify controlled clinical trials leading to widen the licensed indications for this class of therapeutics.

